# Supplementary material for: Hydroclimatic instability accelerated the socio-political decline of the Tang Dynasty in northern China
Source: Commun Earth Environ. 2025 Nov 26;6(1):1003. doi: 10.1038/s43247-025-03038-x (PMC12701842; doi:10.1038/s43247-025-03038-x)
Supplement: Supplementary file 2 — Supplementary Material [file 43247_2025_3038_MOESM2_ESM.pdf]

Supplementary material

Supplementary material to Kempf et al. (2025) *Hydroclimatic instability accelerated the socio-political decline of the Tang Dynasty in northern China* including additional maps and data tables from the material and methods section.

Supplementary Notes 1

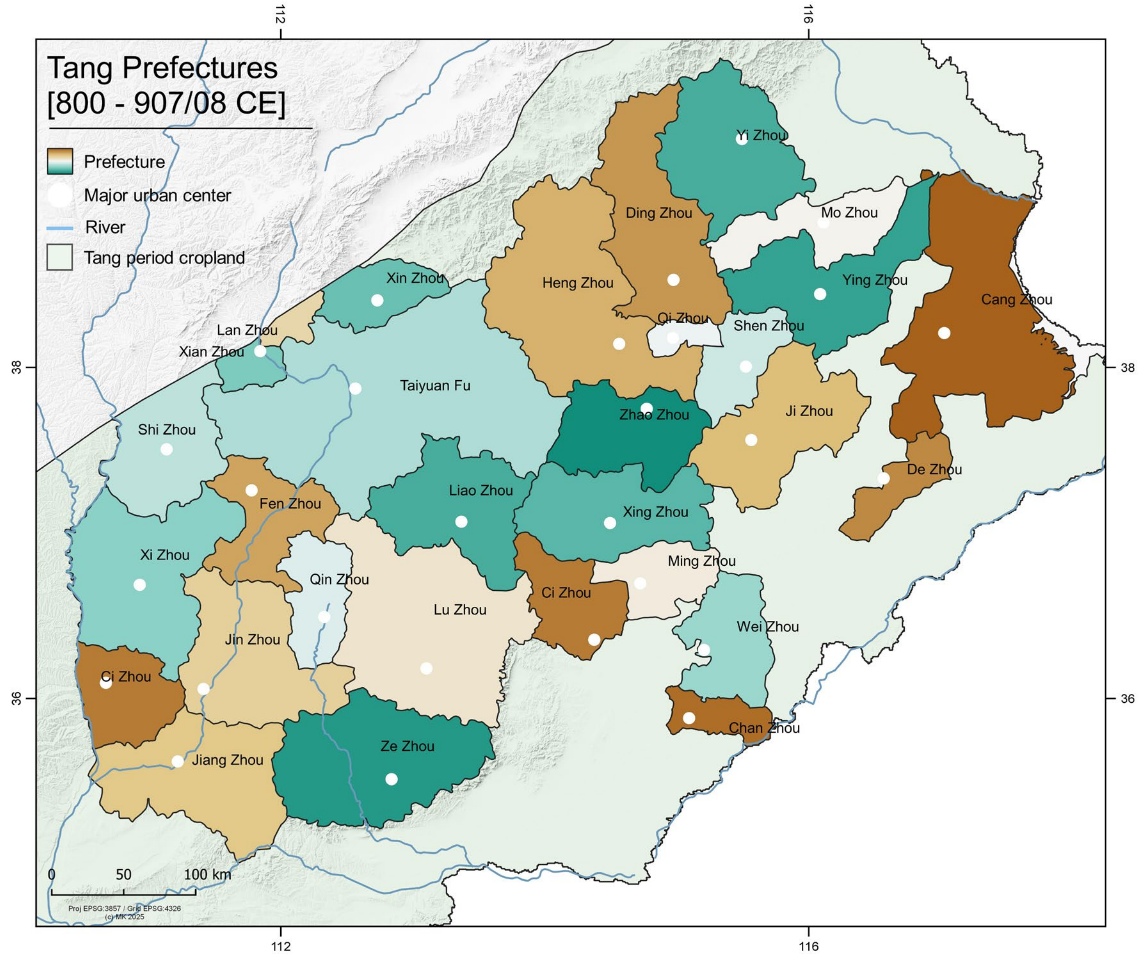

Fig. S1: Prefectures in late Tang agricultural cropland. Tang Dynasty urban centers and prefectures [800-907/908 CE] were extracted from CHGIS[1,2] (last accessed 05th of April 2025). The database was subset according to BEG_YR (start age) and END_YR (end date) for events within the period 800-907/908 CE.

Supplementary Notes 2

Land-use probability components

Five components were used in a combined land-use probability model: multiannual mean temperature; climatic water balance[3], described as precipitation minus potential evapotranspiration; slope; and elevation as well as soil classes based on the FAO classification[4,5]. Soil classes were grouped based on the general classification, e.g., Cambisols. Here, we present both the grouped and the full name small-scale soil mosaic data.

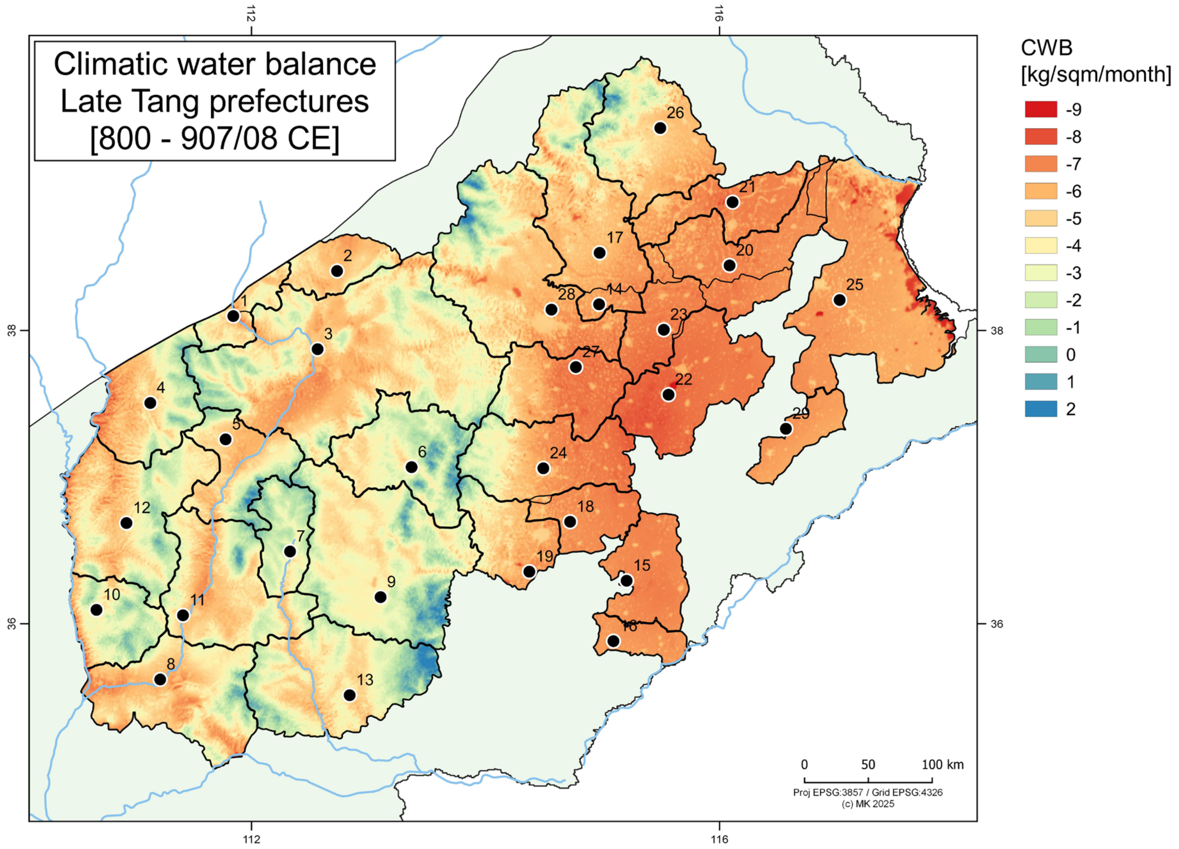

Fig. S2: Multiannual climatic water balance (CWB) (monthly, 1980-2018) cropped to the extent of the late Tang prefectures. Monthly CMI (climatic moisture index=climatic water balance) gridded data derived from CHELSA[3].

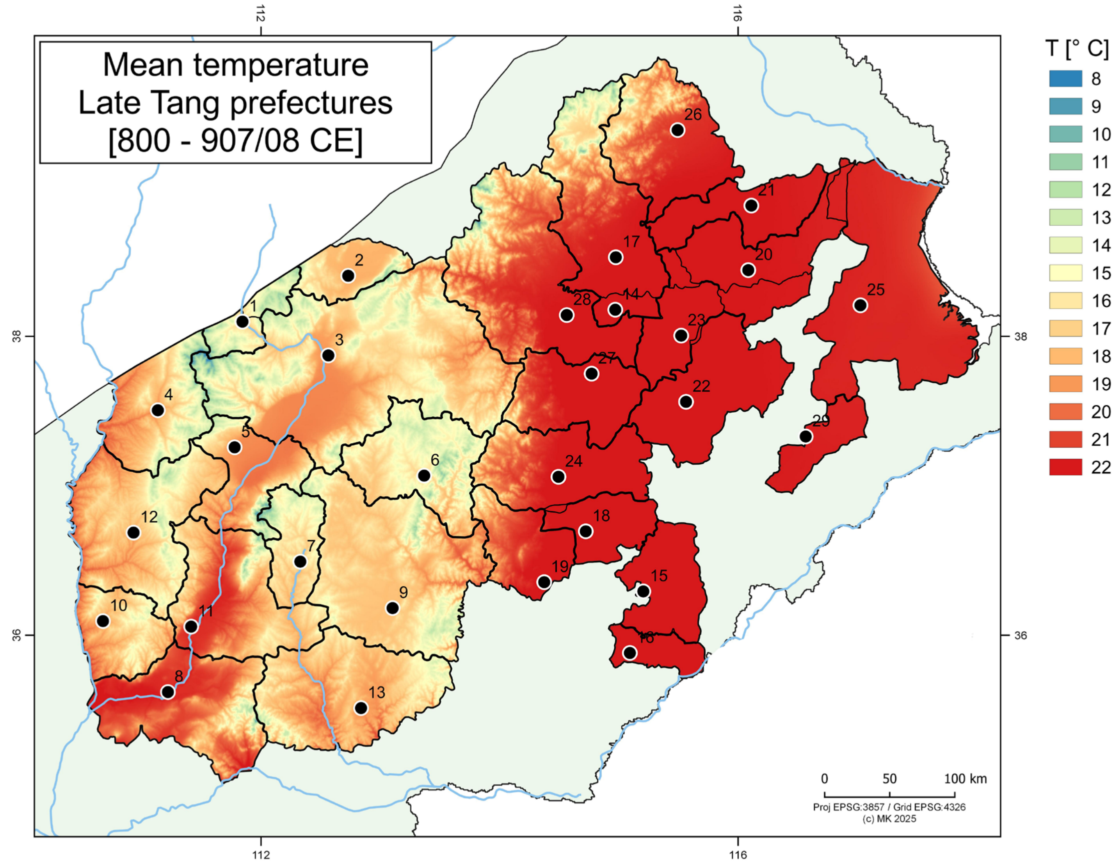

Fig. S3: Multiannual mean temperature (monthly, 1980-2018) cropped to the extent of the late Tang prefectures. Monthly temperature gridded data derived from CHELSA[3].

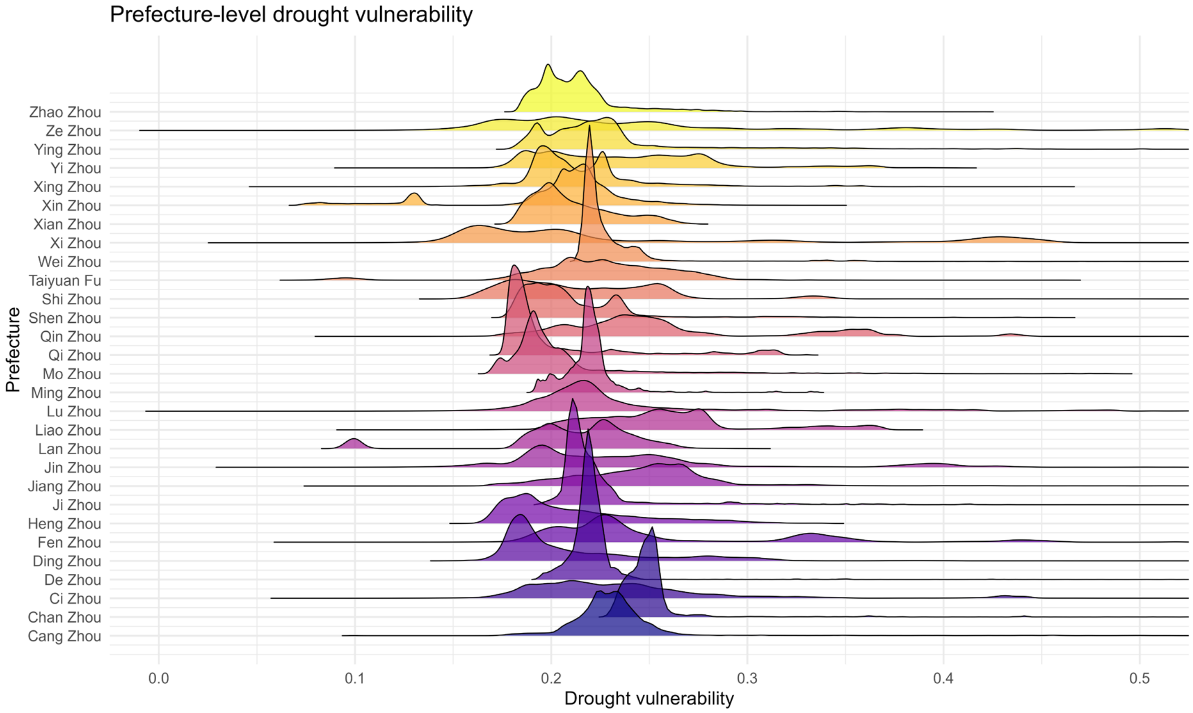

Fig. S4: Prefecture-level drought vulnerability distribution. Frequency of gridded data for each prefecture in late Tang agricultural cropland (see Fig. 2 for the hydroclimatic vulnerability model and Fig. S4 for the extent of the prefectures). High values indicate low drought vulnerability, low values indicate high drought vulnerability.

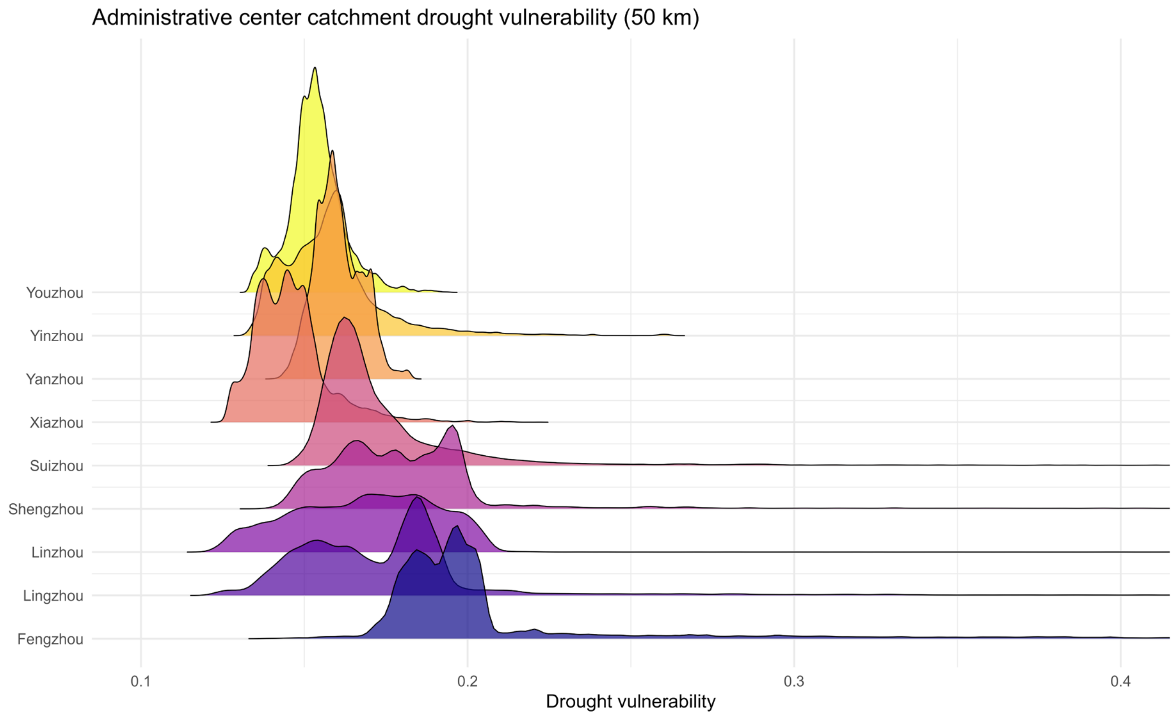

Fig. S5: Administrative center drought vulnerability distribution. Frequency of gridded data for each administrative site in the northern YRL area within a buffer of 50 km. High values indicate low drought vulnerability, low values indicate high drought vulnerability.

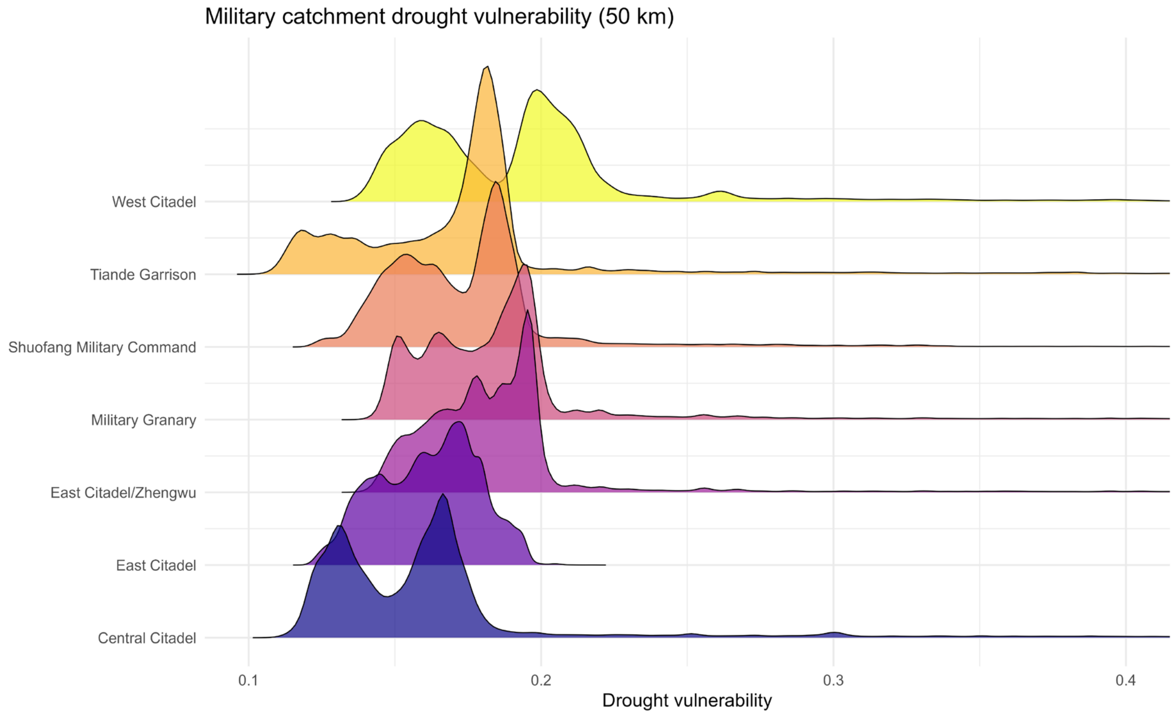

Fig. S6: Military sites drought vulnerability distribution. Frequency of gridded data for each military site in the northern YRL area within a buffer of 50 km. High values indicate low drought vulnerability, low values indicate high drought vulnerability.

Supplementary Notes 3

Hydroclimatic vulnerability components

Meteorological droughts (deficit in precipitation), hydrological droughts (reduction in water supply), and agricultural/ecological droughts (plant water stress due to increased evaporative demand) are strongly interrelated and can cause cascading effects that lead to socio-political and -economic shifts[6]. Components for the hydrological vulnerability model (Fig. 2) are the multiannual SPEI drought index composite (Fig. S7) and the simulated successive flow accumulation (Fig. S8).

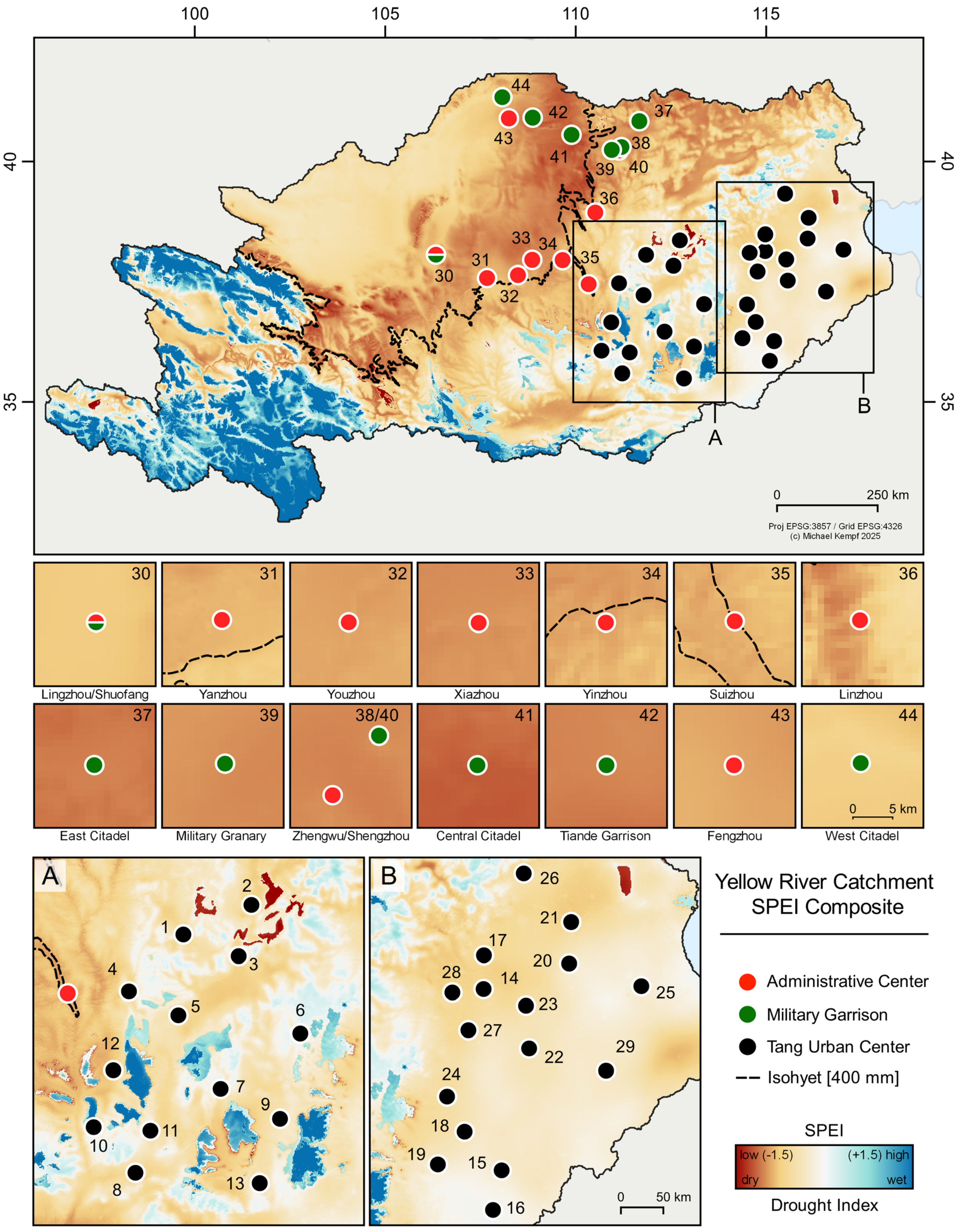

Fig. S7: Gridded multiannual (1980-2018) SPEI index based on 468 monthly gridded data of the climatic water balance.

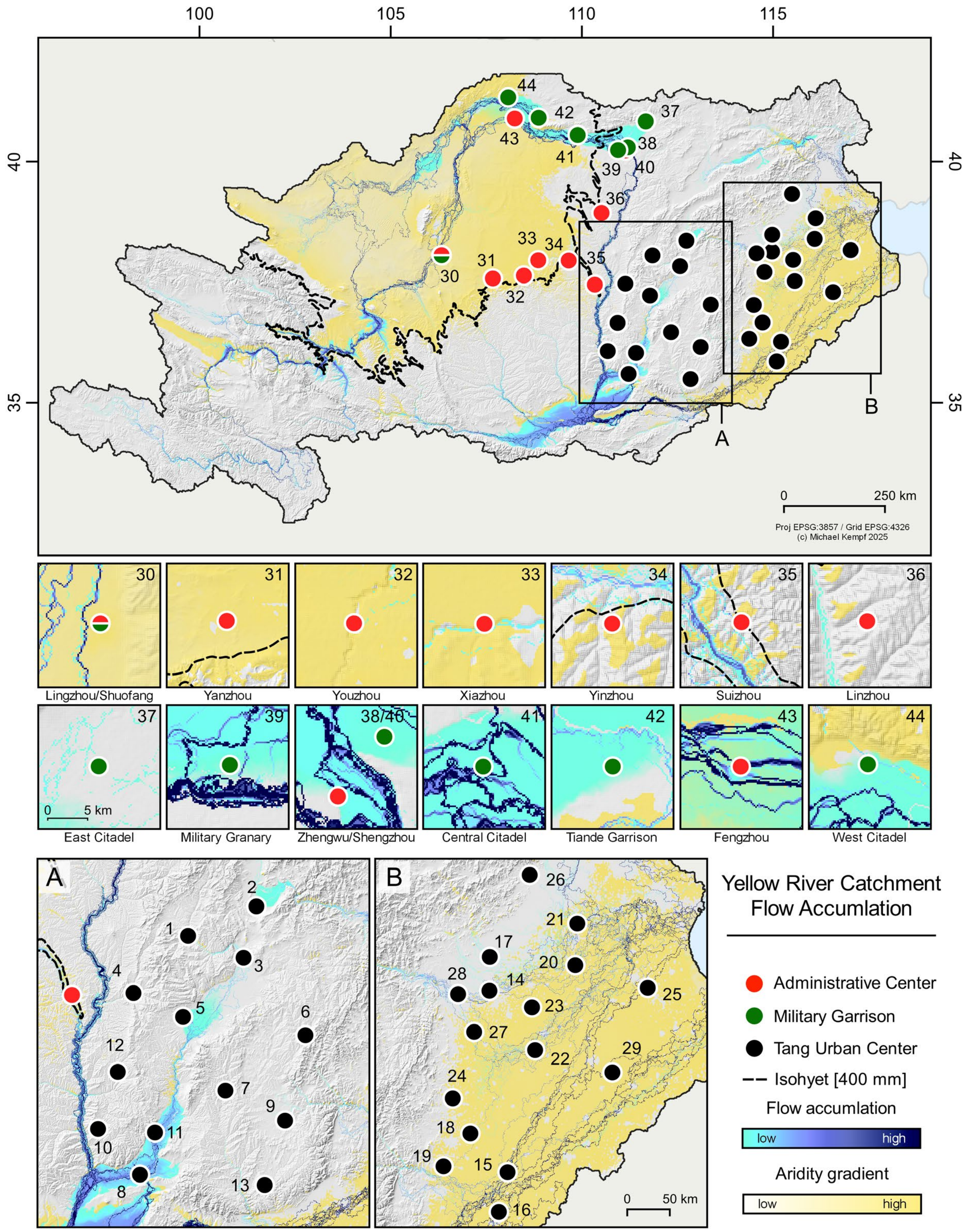

Fig. S8: Successive flow accumulation (iterations = 10). Flooding simulation and major Yellow River branching system based on terrain roughness for each administrative and military site and the Tang urban centers. The aridity gradient highlights the multiannual CWB and a threshold of mean+SD/2 across the catchment.

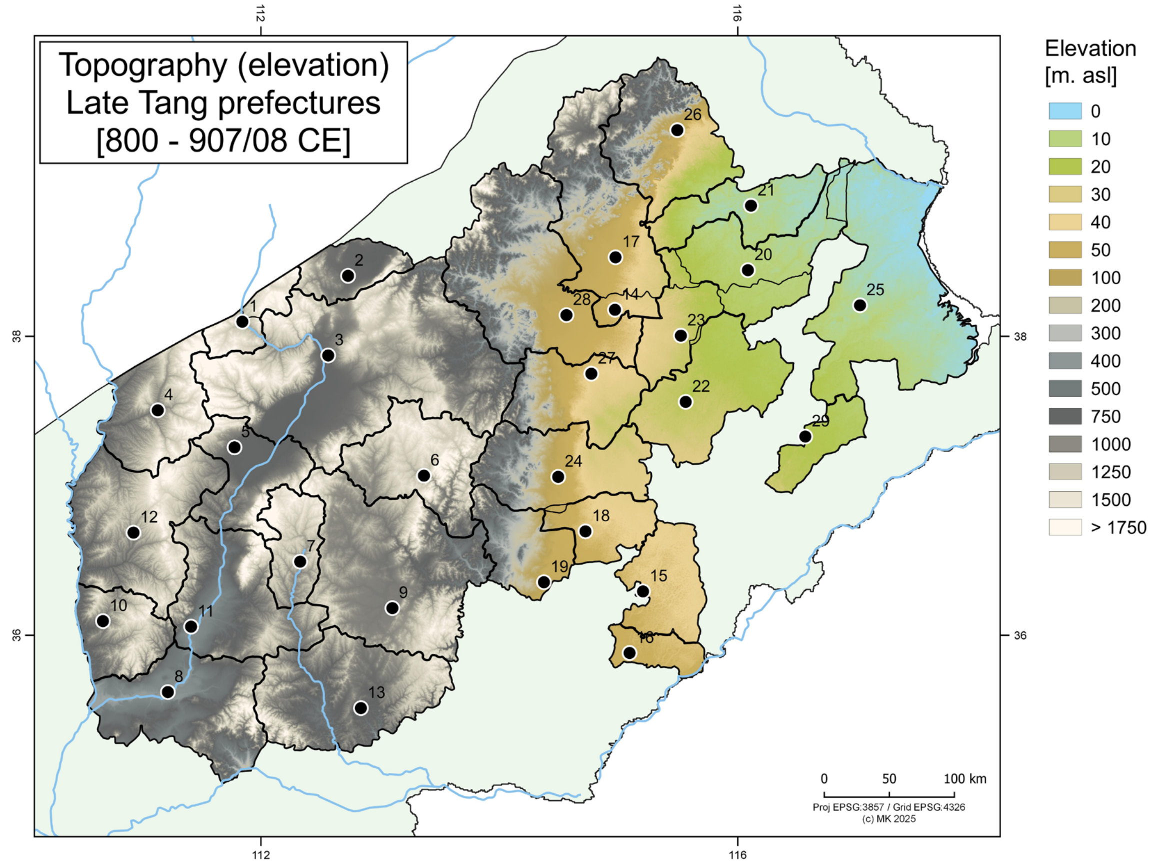

Fig. S9: Late Tang prefecture topography. Elevation in meters above sea level based on the digital elevation model (DEM) cropped to the late Tang prefecture extent. (DEM, USGS: Global Multi-resolution Terrain Elevation Data 2010[7], https://earthexplorer.usgs.gov/; last accessed 15th of January 2025).

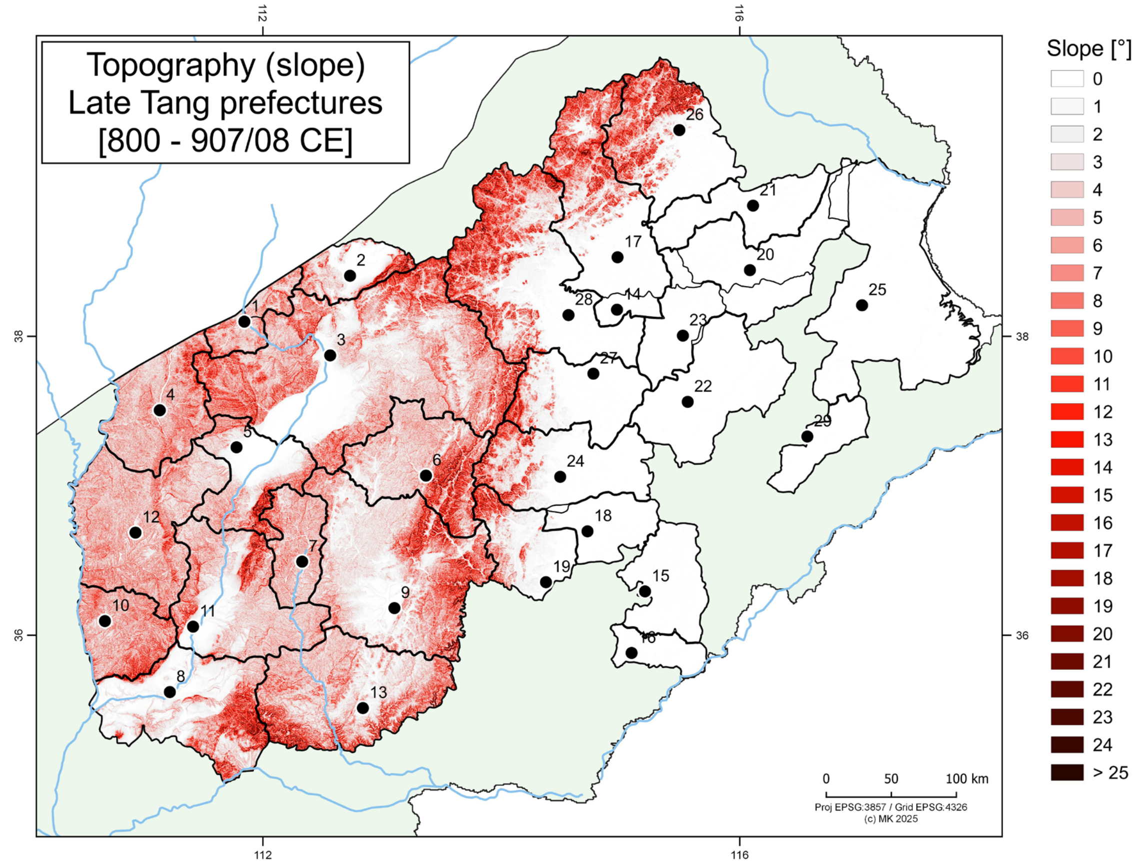

Fig. S10: Late Tang prefecture topography. Slope values in degrees based on the digital elevation model (DEM) cropped to the late Tang prefecture extent. (DEM, USGS: Global Multi-resolution Terrain Elevation Data 2010[7], https://earthexplorer.usgs.gov/; last accessed 15th of January 2025).

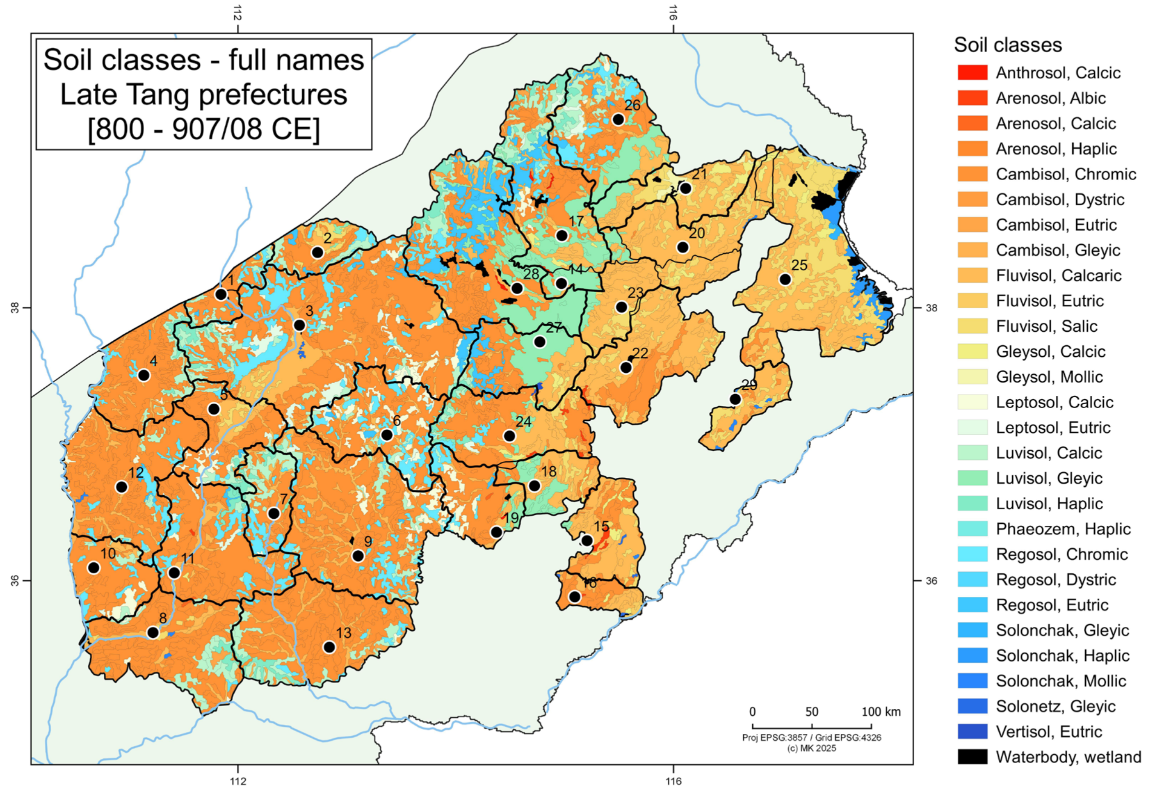

Fig. S11: Late Tang prefecture soil classification. Soil classification (full names) based on the FAO taxonomy[4,5].

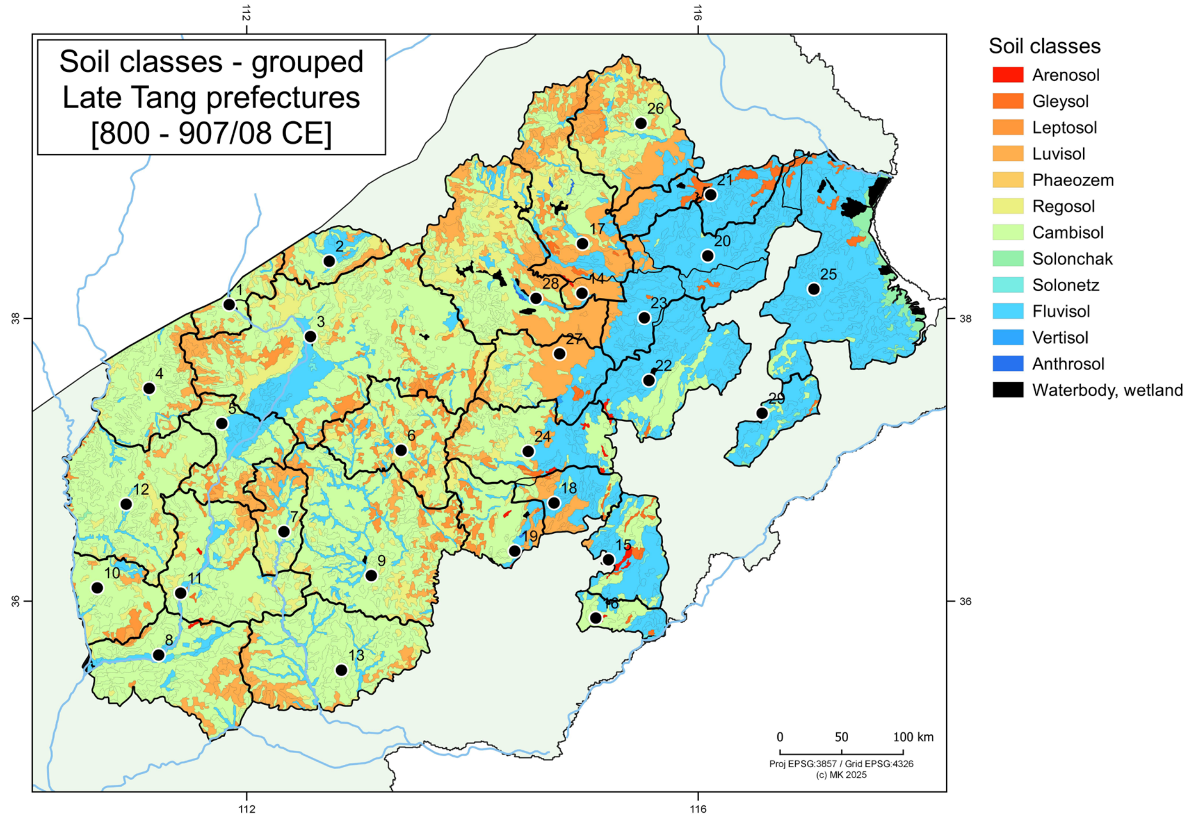

Fig. S12: Late Tang prefecture soil classification. Soil classification (grouped) based on the FAO taxonomy[4,5].

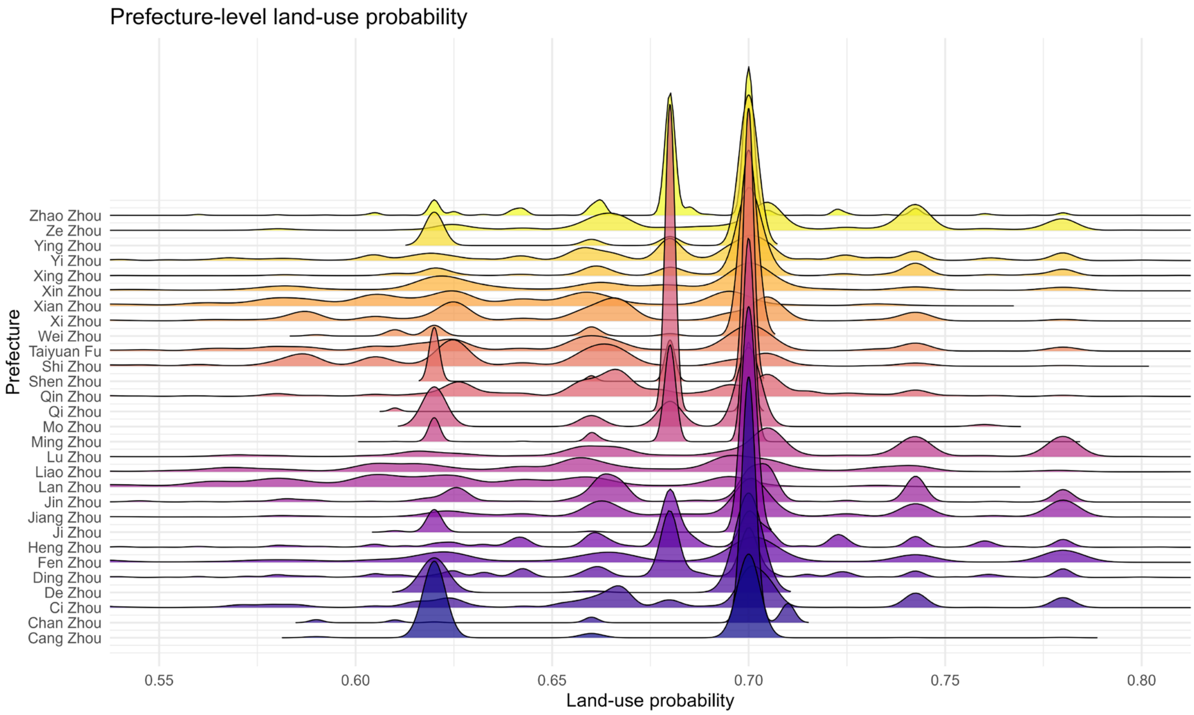

Fig. S13: Prefecture-level land-use probability data distribution.

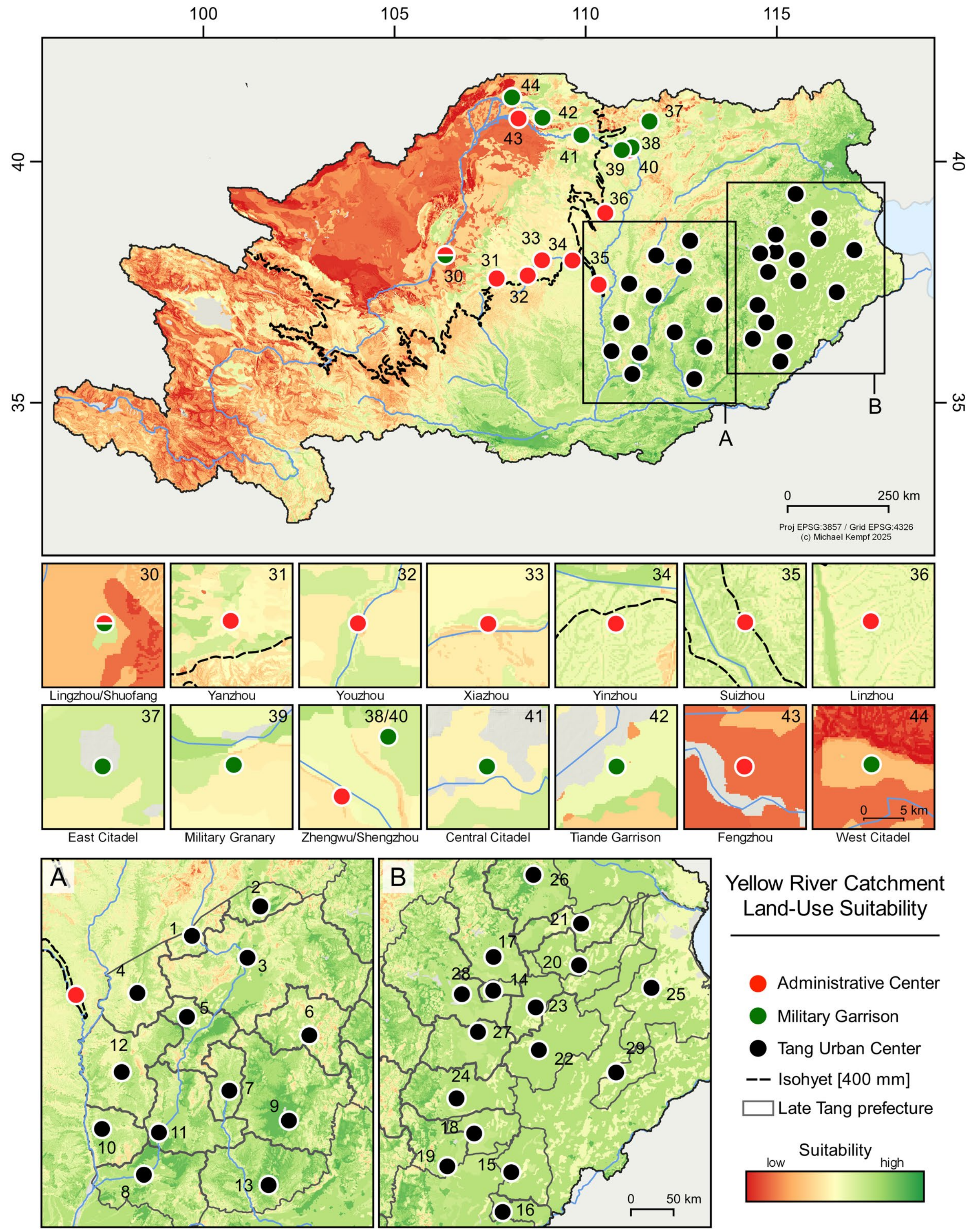

Fig. S14: Land-use suitability and crop production probability across the Yellow River watershed. Land-use potential are the accumulative scores of soil properties, topography and climate variables. Reference period of the climate variables 1980-2018. Tang towns and prefecture data acquired from CHGIS[1,2], last accessed 05th of April 2025 (refer to Fig. 1 and Tab. S1 for site names; see Fig. S13 for data distribution in each prefecture). Grey areas are no-data patches from missing soil data.

Supplementary Notes 4

Connectivity models

We present additional landscape permeability models that center the administrative and military sites of the northern YRL into the broader context of the topographical, hydrological, and climatic conditions of the Yellow River catchment. For this reason, we created the overall administrative and the military site permeability and connectivity for the entire watershed (Figs. S15-S16) . The models show the highest probability movement corridors or pathways while travelling from all across the Yellow River watershed towards each single site. Like this, the connectivity between the sites and the general landscape permeability can be estimated depending on the chosen covariates underlying the model. Here, we used topography (elevation and slope defined as roughness), aridity, and flooding potential as possible drivers of transportation.

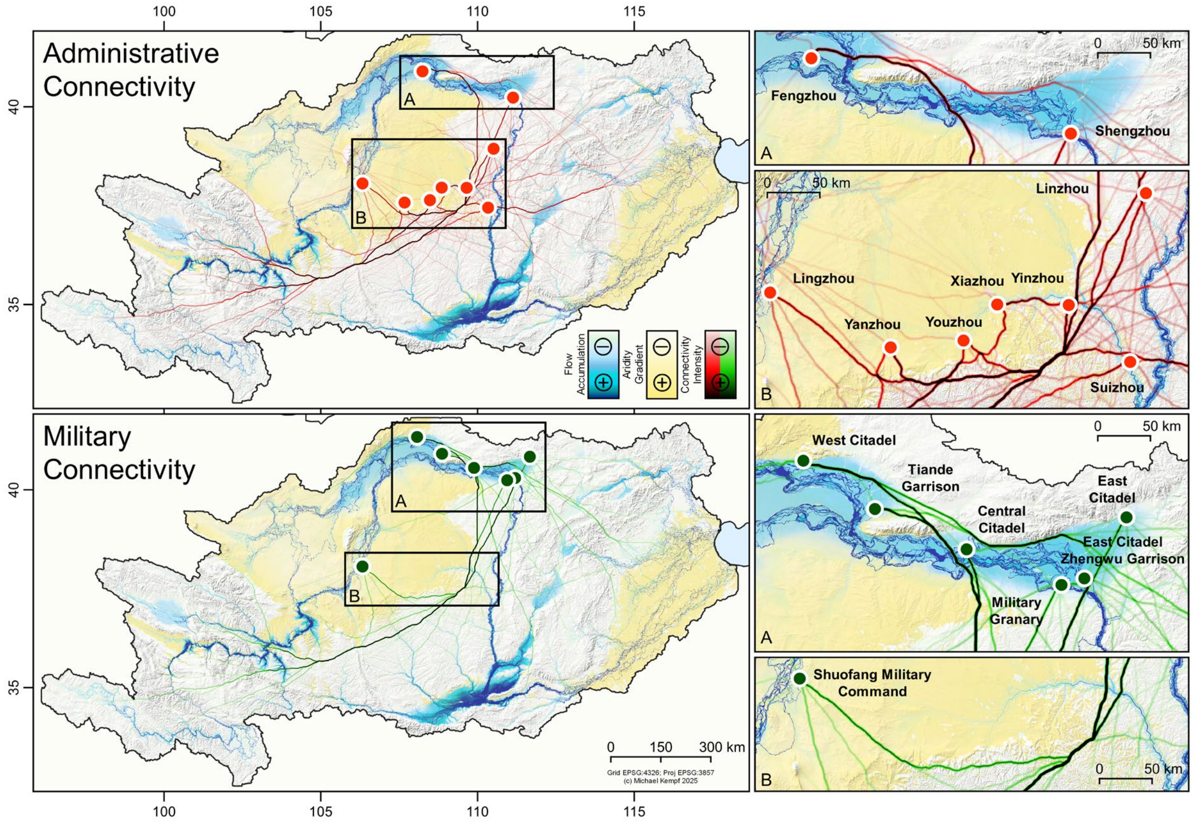

Fig. S15: Connectivity model of Tang Dynasty administrative centers and military garrisons based on topography, flooding vulnerability, and aridity. High density refers to high probability of connectivity and movement corridors.

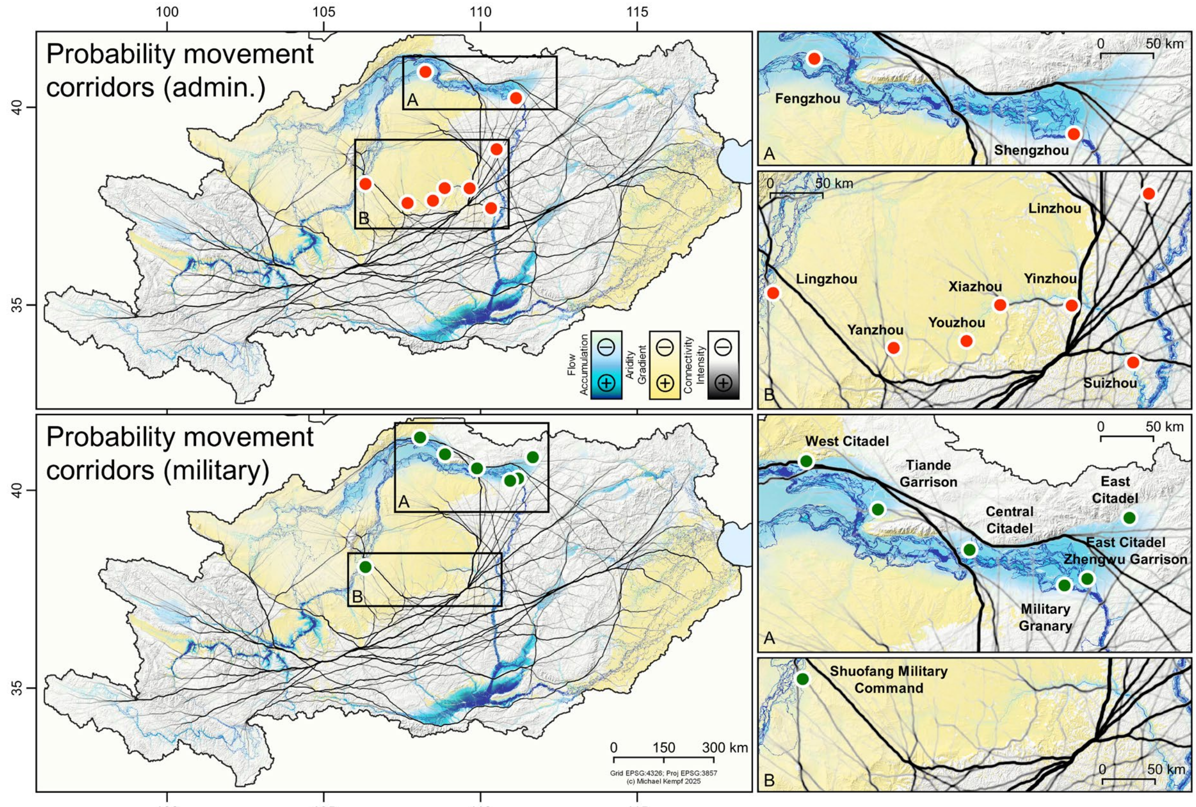

Fig. S16: General landscape permeability and connectivity model in a FETE approach (from everywhere to everywhere) based on topography, flooding potential and aridity threshold.

Supplementary Notes 5

Site names and locations

Table S1 shows the site names and locations as well as the period in which the sites were occupied. Site names and locations were extracted from CHGIS[1,2] (last accessed 05th of April 2025). The database was subset according to BEG_YR (start age) and END_YR (end date) for events occurring within the period 800-907/908 CE. Additional sites for military fortifications and administrative centers were added for the northern part of the YRL.

The 742 census lists the Western Citadel with 7,000 men and 1,700 horses, the Central Citadel with 6,000 men and 2,000 horses, the Eastern Citadel with 7,000 men and 1,700 horses, and the Zhenwu Garrison with 9,000 men and 1,600 horses[8,9]. The reported figure of 30,000 new households is likely exaggerated but still indicative of local population growth during that period[10]. Table S2 shows the components and classifications as well as the scoring system that was applied to create the land-use model.

Tab. S1: Names and locations of administrative centers and military sites used in the analysis. Additional Tang Dynasty urban centers and prefectures [800-907/908 CE] were extracted from CHGIS[1,2] (last accessed 05th of April 2025). The database was subset according to BEG_YR (start age) and END_YR (end date) for events within the period 800-907/908 CE.

**Tang Urban**					
NAME	X	Y	BEG_YR	END_YR	ID
Xian Zhou	111.844743	38.096677	889	1001	1
Xiurong Jun	112.731508	38.398588	410	549	2
Taiyuan Fu	112.565127	37.873765	723	978	3
Xifen Zhou	111.135442	37.510113	552	576	4
Fen Zhou	111.779308	37.263405	526	560	5
Liao Zhou	113.368425	37.07427	623	624	6
Qin Zhou	112.329846	36.497096	596	605	7
Dongyong Zhou	111.21996	35.613583	534	559	8
Shangdang Jun	113.103789	36.183506	-221	-205	9
Nanfen Zhou	110.675417	36.095606	537	549	10
Beiwucheng Jun	111.414989	36.058032	540	560	11
Fen Zhou	110.93169	36.692292	488	525	12
Gai Zhou	112.839451	35.504272	623	626	13
Qi Zhou	114.97085	38.175327	893	1003	14
Wei Zhou	115.205958	36.296328	565	606	15
Chan Zhou	115.093333	35.879416	621	626	16
Xianyu Jun	114.975406	38.52008	581	582	17
Guangping Jun	114.723413	36.701007	284	582	18
Ci Zhou	114.374847	36.35852	590	605	19
Hejian Jun	116.085921	38.435711	387	582	20
Mao Zhou	116.111934	38.85797	711	724	21
Changle Jun	115.564182	37.565976	184	582	22
Shen Zhou	115.523612	38.004602	713	741	23
Xing Zhou	114.494605	37.066343	596	606	24
Cang Zhou	117.026395	38.204597	618	741	25
Yi Zhou	115.493858	39.349074	581	606	26

Zhao Jun	114.772531	37.752927	387	582	27
Changshan Jun	114.563995	38.140121	220	582	28
Ande Jun	116.567386	37.335277	483	583	29
**Admin. Center**					
NAME	X	Y	BEG_YR	END_YR	ID
Lingzhou	106.329554	38.1010655			30
Yanzhou	107.6709293	37.61902159			31
Xiazhou	108.8570566	37.99620121			33
Suizhou	110.3369775	37.49044887			35
Yinzhou	109.6533652	37.99108415			34
Youzhou	108.4818059	37.67964737			32
Linzhou	110.5119562	38.96596489			36
Fengzhou	108.2432954	40.85982865			43
Shengzhou	111.1328332	40.22240796			40
**Military Garrison**					
NAME	X	Y	BEG_YR	END_YR	ID
West Citadel	108.0718428	41.27890312			44
Tiande Garrison/Fengzhou Defense Command	108.8684172	40.87431587			42
Central Citadel	109.8885185	40.53378758			41
East Citadel/Zhengwu Garrison	111.199192	40.28746258			38
East Citadel	111.6686578	40.80361263			37
Military Granary	110.9449914	40.23199701			39
Lingzhou/Shuofang Military Command	106.329554	38.1010655			30

Tab. S2: Soil classification according to FAO taxonomy and land-use probability components and scores. Date from [4,5].

Soil	Score	Elevation	Score	Slope	Score	TMP	Score	CWB	Score
Luvisol, Luvic	0.9	≤1000	1	≤2°	1	<5	0	<-10	0
Luvisol, Haplic	0.85	1000–1500	0.75	2–5°	0.75	5–8	0.5	-10 to -5	0.4
Luvisol, Calcic	0.8	1500–1800	0.4	5–10°	0.5	8–11	0.7	-5 to 0	0.6
Luvisol, Gleyic	0.6	1800–2500	0.1	10–20°	0.2	11–16	0.9	0 to 5	0.8
Kastanozem, Haplic	0.85	>2500	0	>20°	0	16–23	1	5 to 12	1
Kastanozem, Calcic	0.75					23–27	0.8	>12	0.7
Kastanozem, Luvic	0.8					>27	0.6		
Cambisol, Eutric	0.8								
Cambisol, Chromic	0.7								
Cambisol, Dystric	0.5								
Cambisol, Gleyic	0.5								
Cambisol, Humic	0.6								
Cambisol, Umbric	0.55								
Fluvisol, Eutric	0.75								
Fluvisol, Calcaric	0.7								
Fluvisol, Salic	0.3								
Chernozem, Haplic	1								
Chernozem, Calcic	0.85								
Chernozem, Luvic	0.9								
Vertisol, Eutric	0.7								
Gypsisol, Haplic	0.4								
Gypsisol, Calcic	0.4								
Gypsisol, Luvic	0.45								
Arenosol, Haplic	0.3								
Arenosol, Calcic	0.35								
Arenosol, Albic	0.25								
Solonchak, Calcic	0.2								
Solonchak, Gleyic	0.15								
Solonchak, Mollic	0.3								

Solonchak, Gypsic	0.2								
Solonchak, Haplic	0.3								
Solonchak, Natric	0.2								
Solonetz, Calcic	0.2								
Solonetz, Gleyic	0.15								
Leptosol, Calcic	0.25								
Leptosol, Eutric	0.3								
Leptosol, Mollic	0.35								
Leptosol, Humic	0.35								
Leptosol, Gypsic	0.25								
Greyzem, Haplic	0.6								
Phaeozem, Haplic	0.9								
Phaeozem, Gleyic	0.7								
Planosol, Eutric	0.5								
Regosol, Eutric	0.6								
Regosol, Chromic	0.5								
Regosol, Dystric	0.4								
Anthrosol, Calcic	0.7								
Anthrosol, Ferric	0.65								
Anthrosol, Albic	0.6								
Gleysol, Eutric	0.5								
Gleysol, Calcic	0.5								
Gleysol, Mollic	0.6								
Gleysol, Humic	0.55								
Histosol, Sapric	0.4								
Podzol, Dystric	0.2								

Supplementary References

1. CHGIS. V6 Time Series Prefecture Polygons. 31098883, 30720236, 1093219, 30722275, 31096825, 31096134, 30719545, 31098173, 30721584 Harvard Dataverse https://doi.org/10.7910/DVN/I0Q7SM (2016).
2. CHGIS. V6 Time Series Prefecture Points. 282615, 288343, 288578, 282850, 4937707 Harvard Dataverse https://doi.org/10.7910/DVN/WW1PD6 (2016).
3. Karger, D. N. *et al.* Climatologies at high resolution for the earth's land surface areas. *Sci. Data* **4**, 170122 (2017).
4. Dijkshoorn, J. A., van Engelen, V. W. P. & Huting, J. R. M. *Soil and Landform Properties for LADA Partner Countries (Argentina, China, Cuba, Senegal and The Gambia, South Africa and Tunisia). ISRIC Report 2008/06 and GLADA Report 2008/03, ISRIC – World Soil Information and FAO*. http://www.isric.org/isric/Webdocs/Docs/ISRIC_Report_2008_06.pdf (2008).
5. IUSS Working Group WRB. *World Reference Base for Soil Resources: International Soil Classification System for Naming Soils and Creating Legends for Soil Maps. 4th Edn, International Union of Soil Sciences (IUSS)*. https://www.isric.org/sites/default/files/WRB_fourth_edition_2022-12-18.pdf (2022).
6. Intergovernmental Panel On Climate Change (Ipcc). *Climate Change 2022 – Impacts, Adaptation and Vulnerability: Working Group II Contribution to the Sixth Assessment Report of the Intergovernmental Panel on Climate Change*. (Cambridge University Press, 2023). doi:10.1017/9781009325844.
7. Earth Resources Observation and Science (EROS) Center. Global Multi-resolution Terrain Elevation Data 2010 (GMTED2010). U.S. Geological Survey https://doi.org/10.5066/F7J38R2N (2010).
8. Du, Y. *Comprehensive Statutes*. (Zhonghua Shuju, Beijing, 1988).
9. Wang, P. *Essential Documents of the Tang*. (Shanghai Guji Chubanshe, Shanghai, 1991).
10. Li, J. *Treatise on the Administrative Units of the Yuanhe Era, 806–820*. vol. 468 (Shanghai Guji Chubanshe, Shanghai, 1993).
